# Normothermic Machine Perfusion in Orphan Liver Graft Viability Assessment

**DOI:** 10.3390/jcm14030777

**Published:** 2025-01-24

**Authors:** Marcin Morawski, Andriy Zhylko, Hubert Kubiszewski, Jakub Rochoń, Paweł Rykowski, Mikołaj Staszewski, Maciej Krasnodębski, Wojciech Figiel, Marek Krawczyk, Michał Grąt

**Affiliations:** Department of General, Transplant and Liver Surgery, Medical University of Warsaw, 02-097 Warsaw, Poland; zhylko.andrey@gmail.com (A.Z.); hubertkubiszewski23@gmail.com (H.K.); kuba.rochon@gmail.com (J.R.); pawel.rykowski@wum.edu.pl (P.R.); mst.staszewski@gmail.com (M.S.); mwkrasn@gmail.com (M.K.); w.figiel@yahoo.es (W.F.); marek.krawczyk@wum.edu.pl (M.K.); michal.grat@wum.edu.pl (M.G.)

**Keywords:** liver transplantation, normothermic machine perfusion, liver machine perfusion, orphan livers

## Abstract

**Background**: Liver transplantation constitutes a well-established treatment for patients with end-stage liver disease and selected hepatic malignancies. The introduction of normothermic machine perfusion (NMP) offers a platform for both extracorporeal organ maintenance and viability assessment, especially for organs with suspicious malfunction. These organs, discarded by the majority of transplant centers (so-called ‘orphan livers’), may help to safely expand the donor pool thanks to pre-transplant appraisal; **Methods**: We identified all grafts undergoing normothermic ma-chine perfusions performed in the Department of General, Transplant, and Liver Surgery between December 2022 and August 2023. Their perfusion characteristics and immediate postoperative periods, as well as complications that occurred in the 90-day postoperative periods, were analyzed; **Results**: There were eight orphan liver grafts that underwent NMP in our Department. Postoperative complications occurring in patients receiving grafts after NMP did not seem associated with the procedure. One patient required laparotomy within the 90-day postoperative period due to biliary fistula and underwent bile duct stenting due to both fistula and nonanastomotic stricture. In one patient we observed the occurrence of anastomotic biliary stricture more than 90 days after LTx; **Conclusions**: NMP allows for the viability assessment of grafts with suspicious prepreservation malfunction. Some of these organs may help to expand the donor pool.

## 1. Introduction

Liver transplantation (LTx) constitutes a well-established treatment for patients with end-stage liver disease and selected hepatic malignancies [1]. An insufficient number of organ donors remains the most important limitation of the method and contributes to waiting-list mortality. The utilization of grafts procured from extended criteria donors (ECD) helps to increase the pool of available organs. However, this group of organs is particularly susceptible to ischemia-reperfusion injury (IRI) resulting in poor graft function and patient morbidity [2]. Thus, alleviation of IRI seems an important target to be addressed by developing novel strategies of organ preservation, especially in the era of increasing numbers of ECD. There are two widely used protocols for machine perfusion of the liver—dual hypothermic oxygenated machine perfusion (dHOPE) and normothermic machine perfusion (NMP).

The HOPE protocol is based on the dual arterial and portal perfusion of an oxygenated preservation solution at a temperature of 12 degrees Celsius. This method has been shown to alleviate sequelae of IRI by improving mitochondrial function. The favored low-flux electron transfer, which protects against reactive oxygen species accumulation, as well as the increased adenosine triphosphate content lead to lower apoptosis and necrosis rates and mitigate inflammatory response [3]. It has been recently shown in a randomized trial performed by our group that application of HOPE may be beneficial in high-risk donors after brain death (a Donor Risk Index of more than 1.70 [4] significantly reduces the Model for Early Allograft Function (MEAF) score). The results of other randomized studies also show the benefits of hypothermic perfusion with respect to both clinical endpoints and surrogates of early allograft function [5,6,7].

In NMP, the circulating fluid is based on a banked packed red blood concentrate, which is oxygenated, rewarmed to 37 °C, and supplemented with electrolytes, nutrients, and vitamins [8]. As opposed to the HOPE protocol, NMP not only does mitigate sequelae of IRI but also allows for graft viability assessment [9]. Thanks to the restoration of physiologic temperature and provision of nutrients, cell viability can be assessed by observing acid–base balance, lactate clearance, glucose uptake, and bile production [9]. Since the validation of NMP in clinical settings in 2013, there have been three randomized trials on NMP [8,10,11,12]. A significant reduction in early aminotransferase activity has been observed in the NMP arm by Nasralla et al. [10]. Ghinolfi et al. failed to show any clinical benefits of NMP, yet histopathological specimens were significant for evidence of reduced IRI [8]. A recent study by Markmann et al. showed favorable short- and long-term results (reduced early allograft dysfunction and biliary complications) as well as justified higher use of livers from donors after cardiac death [12].

Data on the safety of usage of orphan livers following NMP are scarce [13]. In the presented study, we report on the application of NMP in cases of livers with suspicious prepreservation malfunction (so called “orphan livers”) [14].

## 2. Materials and Methods

Herein, we present a retrospective analysis of the first eight cases of NMP performed in our department. In the period between December 2022 and August 2023, all livers offered to the Department of General, Transplant, and Liver Surgery were screened for eligibility. Only grafts discarded by all of the national transplant centers with presumed pre-preservation dysfunction (elevation in International Normalized Ratio [INR] and elevated aminotransferase activity) and known prepreservation injury were included in the study and underwent viability assessment by NMP. None of the livers offered to our department with suspected dysfunction were excluded from the study. The NMP program was commenced in the department after the successful implementation of the dHOPE procedure, which was recently summarized in papers by Grąt et al. and Morawski et al. [15,16]. The study plan was reviewed by the Institutional Review Board of the Medical University of Warsaw (AKBE/266/2023). Due to the retrospective nature of the study, consent to participate was waived.

**Figure 1:**
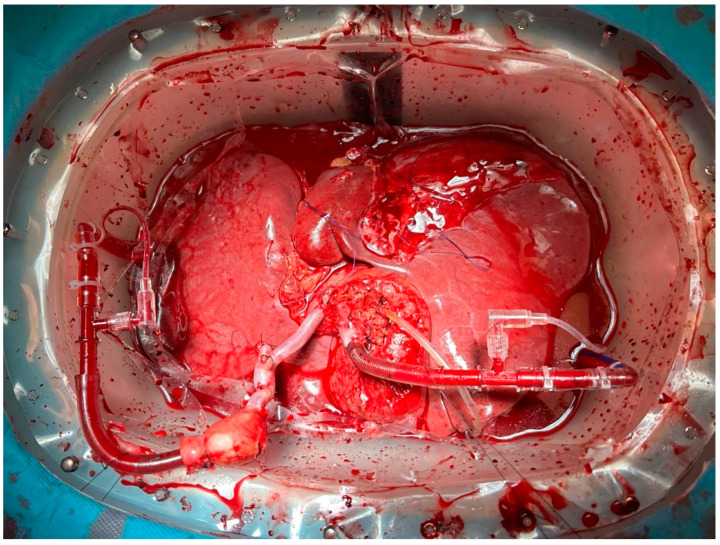
Example of end-ischemic normothermic machine perfusion through the hepatic artery and portal vein in the Department of General, Transplant and Liver Surgery.

The liver grafts, after back-table preparation, underwent normothermic machine perfusion using a Liver Assist device (XVIVO, Groningen, the Netherlands) (Figure 1). In all but one of the cases, a packed RBC-based perfusate was used according to the protocol by Ghinolfi et al. [8]—(1) ABO-compatible red blood cell concentrate (750 mL); (2) Gelofusine^®^ (Braun, Melsugen, Germany) (1000 mL); (3) human albumin 200 g/L (100 mL); (4) parenteral nutrition (10 mL); (5) multivitamin intravenous solution (10 mL); (6) metronidazole intravenous solution 5 mg/mL (40 mL); (7) cefazolin 1 g (10 mL); (8) fluconazole 100 mg (50 mL); (9) fast acting insulin 100 UI/mL (20 mL); (10) calcium gluconate intravenous solution 10% (40 mL); (11) sterile water (50 mL); (12) sodium chlorate 0.9% solution (150 mL); (13) sodium bicarbonate 8.4% solution (30 mL); (14) heparin 25,000 units (30 mL). In case 1, a donor whole blood-based perfusate supplemented with insulin, sodium bicarbonate, heparin, antibiotics (cefazolin, metronidazole, and fluconazole), and electrolytes was used. The temperature of the perfusate was initially set at 20 °C and slowly warmed up to 37 °C (approximately one degree every two minutes). A concentration of 100% oxygen was supplied to the oxygenator, and the perfusion pressures were set at 90 mm Hg for the arterial line (pulsatile flow) and 9 mm Hg for the portal line (continuous flow). Perfusate samples were taken at the beginning and every 30 min afterwards for blood gas analysis (ABL 800 Flex, Radiometer, Copenhagen, Denmark). After one and two hours of perfusion, a sample of perfusate was collected and activity of aminotransferases was measured. Prior to transfer to the operating table, the liver was flushed through the hepatic artery and portal vein with 2 L of cold preservation solution (StoreProtect Plus, Carnamedica, Warszawa, Poland). Until the completion of caval anastomosis, the allograft was continuously flushed with cold 0.9% saline. One discarded liver (case 5) underwent dual hypothermic oxygenated perfusion (dHOPE) prior to NMP.

The viability assessment performed during the two-hour perfusion justified utilization of the graft. The main parameter of interest was lactate clearance within the first hour of perfusion (measured at 30 min and 1 h after initiating NMP). We also adopted the criteria for hepatocyte viability recently presented by van Leeuwen et al. [17]—(1) a perfusate pH of 7.35–7.45 (green zone) or 7.25–7.35 (orange zone), (2) a perfusate lactate of less than 1.7 mmol/L (green zone) or less than 4.0 mmol/L (orange zone), and (3) bile production of more than 10 mL (green zone) or between 5 and 10 mL (orange zone) (Table 1). In addition, the livers were assessed macroscopically and by palpation (parenchyma firmness during perfusion).

The perfusate was supplemented with electrolytes and bicarbonate as needed. During the first two hours of perfusion, all but one liver required frequent potassium correction. Bicarbonate was added in case of acidosis and bicarbonate depletion in blood gas analysis.

## 3. Results

The donor and perfusion characteristics are summarized in Table 1. The mean NMP time was 315 min for all livers and 363 min for utilized grafts. The decision on organ acceptance was based on blood gas analyses and biochemical parameters after a 2-h perfusion for all grafts. All of the liver grafts met the criterion of bile production (green zone) secreting more than 10 mL of bile during the first two hours and maintaining this state afterwards. In the first case only, we observed a lactate concentration of 6.2 mmol/L (red zone); however, it was preceded by rapid clearance from 14.0 mmol/L within 30 min.

Additionally, in case 1, the perfusate was supplemented with two units of packed RBCs due to initial low haemoglobin of 4.8 g/dL. The acid–base balance was satisfactory in the first four cases, and cases 3 and 4 achieved “green-zone” results of 7.414 and 7.357, respectively. Interestingly, we observed that the main mechanism that may underline initial correction of acidosis can be attributed to potassium–hydrogen shifts in the perfusate. In all but one of the cases (case 8), we observed a rapid decline in potassium concentration accompanied by an increase in perfusate pH. The only liver that did not present this phenomenon performed poorly during the perfusion (a slow decline in lactates and severe persistent acidosis of less than 6.9 during the first hour). The histopathology of the graft was notable for 20% necrosis of hepatocytes.

### 3.1. Case 1

Donor: a 22-year-old male donor after brainstem death (DBD) due to head trauma with severe hemodynamic instability (noradrenaline of 2.13 μg/mg/min) and elevated liver function tests (ALT 635 U/L; AST 1494 U/L; INR 2.32). Cold ischemia time (CIT) was 200 min. Graft macrosteatosis was estimated to be <5% of hepatocytes.

Recipient: a 41-year-old male with primary sclerosing cholangitis, cirrhosis, and a history of recurrent cholangitis. The patient underwent LTx with piggy-back cavo-caval anastomosis and end-to-end biliary anastomosis. The postoperative period was notable for the significant elevation of aspartate and alanine aminotransferases (AST 14980 U/L, ALT 4188 U/L)—the patient underwent relaparotomy on postoperative day 1 and portal vein reanastomosis due to kinking. The patient was discharged home on postoperative day 21.

### 3.2. Case 2

Donor: a 45-year-old male DBD donor after a cerebrovascular accident with impaired liver function (INR 3.44). Cold ischemia time (CIT) was 260 min. Graft macrosteatosis was estimated at 5% of hepatocytes.

Recipient: a 66-year-old male with alcoholic cirrhosis and hepatocellular carcinoma (HCC) (Child–Pugh A7, MELD 9). The patient underwent LTx with end-to-end caval anastomosis, a veno-venous bypass, and end-to-end biliary anastomosis. In the postoperative period, the patient required hemodialysis due to kidney injury. The patient was discharged home on postoperative day 14. He was readmitted because of biliary stricture that required endoscopic management and bile duct stenting. The stricture was diagnosed > 90 days after surgery.

### 3.3. Case 3

Donor: a 34-year-old male DBD donor after a cerebrovascular accident with impaired liver function (INR 2.06) and a history of alcohol consumption. Cold ischemia time (CIT) was 225 min. Graft macrosteatosis was estimated at 30% of hepatocytes.

Recipient: a 62-year-old male with alcoholic cirrhosis with HCC, portal hypertension (a history of variceal bleeding; a present portosystemic shunt), portal vein thrombosis (Yerdel grade I), recurrent episodes of hepatic encephalopathy, and multivessel coronary disease (after a coronary artery bypass grafting). The patient underwent LTx with piggy-back and end-to-end biliary anastomosis. Severe hypotension following reperfusion occurred, for which the patient required dual vasopressor administration (noradrenaline and adrenaline). Multiorgan failure also occurred due to metabolic acidosis, hypoglycemia, dialysis. The patient received urgent retransplantation on postoperative day 1 and died on postoperative day 1, after reLTx, due to cardiac arrest.

### 3.4. Case 4

Donor: a 66-year-old male DBD donor after asphyxiation and out-of-hospital cardiac arrest with obesity (BMI 37.6) and elevated aminotransferases (ALT 152 U/L, AST 393 U/L). Cold ischemia time (CIT) was 360 min. Graft macrosteatosis was estimated at 15% of hepatocytes.

Recipient: a 51-year-old male with decompensated cryptogenic cirrhosis, severe encephalopathy, ascites, and portal hypertension (referred to the department due to gastrointestinal bleeding, hepato-renal syndrome, and hemodynamic instability). The patient was shortlisted for urgent LTx and transferred to the operating theater on day 2 following admission. Intraoperatively persistent bleeding from the retroperitoneal space occurred after hepatectomy with significant blood loss and multiple transfusions. The LTx was completed with abdominal packing. The patient died on postoperative day 1 due to multiple organ failure and subsequent cardiac arrest.

### 3.5. Case 5

Donor: a 43-year-old female DBD donor after asphyxiation (severe asthma) and cardiac arrest. Cold ischemia time (CIT) was 470 min. Graft macrosteatosis was estimated to be <5% of hepatocytes.

Recipient: a 46-year-old female with polycystic liver disease. The patient underwent LTx piggy-back and end-to-end biliary anastomosis. The patient was discharged home on postoperative day 11. She was readmitted a month later due to abdominal fluid collection and underwent urgent relaparotomy and drainage due to biliary fistula. After surgery, she also had endoscopic recurrent cholangiopancreatography and bile duct stenting due to both biliary fistula and stricture above the anastomosis.

Complications in the postoperative period and the Clavien–Dindo classifications of all the recipients are summarized in Table 2. In one patient, we observed the occurrence of anastomotic biliary stricture more than 90 days after LTx. The patient required endoscopic retrograde cholangiopancreatography and bile duct stenting.

## 4. Discussion

Normothermic machine perfusion offers a valuable tool for the assessment of graft viability prior to LTx and allows for prolonged extracorporeal organ maintenance. In this report, we show our first experience with NMP, which was performed on 8 liver donors that presented with suspicious malfunction or significant prepreservation injury. Based on the functional status of the grafts, we decided to transplant 5 livers following NMP. The decision to transplant was based mainly on the maintenance of perfusate acid–base balance and lactate clearance. Aminotransferase activity was used to evaluate the extent of hepatocyte injury, and its elevation corresponded with poor graft function in blood gas analysis (cases 5, 7, and 8). We also observed significant potassium and hydrogen shifts, which happen early during the perfusion (rapid potassium depletion and pH correction). They restore electrostatic balance across cell membranes and may, in case of their absence, herald vast hepatocyte injury and poor graft function during NMP.

All complications listed in Table 1 that occurred in the postoperative period can hardly be linked to the application of NMP. In case 3, following reperfusion, severe hypotension caused the patient’s rapid deterioration and the development of multiorgan failure. Due to suspected primary nonfunction, the patient was shortlisted for urgent reLTx and underwent the procedure on postoperative day 1. Similarly, during the second LTx, reperfusion was followed by cardiovascular collapse and death in the immediate postoperative period. The intraoperative events corresponded with his known high cardiovascular risk and history of multivessel coronary disease. Case 4 was shortlisted for rescue LTx due to severe decompensation of liver function, coagulopathy, encephalopathy, and recurrent esophageal bleeding. In one of the elective cases, we observed postreperfusion syndrome (PRS), which, in case of NMP, may be linked to mean pO2 and corresponds with the importance of maintenance of oxygen partial pressure between 150 and 190 mmHg, thus minimizing the risk of PRS and vasoplegia [18].

The criteria for hepatocyte and biliary cell viability presented by van Leeuwen offer a valuable tool in decision-making regarding the utilization of grafts during NMP [17]. However, the so-called “orange zone” of organ acceptance provides room for adopting a more personal approach in organ assessment based on the experience of the perfusionist. It seems clear that dynamics of acid–base restoration, ionic shifts, and the macroscopic appearance of the liver may deliver valuable information on graft performance in case of “orange” results of critical parameters.

Since the validation of NMP in clinical settings in 2013, there have been three randomized trials on NMP [8,10,11,12]. A significant reduction in early aminotransferase activity has been observed in the NMP arm by Nasralla et al. [10]. Ghinolfi et al. failed to show any clinical benefits of NMP, yet histopathological specimens were significant for evidence of reduced IRI [8]. A recent study by Markmann et al. showed favorable short- and long-term results (reduced early allograft dysfunction and biliary complications) as well as justified higher use of livers from donors after cardiac death [12]. However, the question of routine use of NMP has not been addressed in the literature yet. In addition, two of the trials reporting on NMP commenced at the donor hospital, whereas data on end-ischemic (immediately prior to transplantation) NMP are scarce [8,12].

## 5. Conclusions

NMP may allow the viability assessment of grafts with presumed prepreservation malfunction. Some of these organs may help to expand the donor pool. More data are needed to establish the impact of the use of suspected dysfunctional livers after viability confirmation by NMP on posttransplant outcomes.

## Figures and Tables

**Table 1:** Summary of normothermic perfusion characteristics in the moment of decision upon organ utilization. ALT—alanine aminotransferase, AST—aspartate aminotransferase, DRI—Donor Risk Index, PRS—post-reperfusion syndrome.

Lp.	Patient	DRI	Perfusion Time	Perfusate	pH	Lactate [mmol/L]			Bile > 10 mL	AST 1 h [U/L]	AST 2 h [U/L]	Mean pO_2_	Mean pCO_2_	LTx	PRS
						30 min	1 h	2 h							
1	1	1.075	7 h 30 min	donor whole blood	7.569	13.5	14.1	6.2	Yes	2763	1457	380	44	Yes	No
2	2	1.262	5 h 30 min	packed RBC-based	7.285	7.7	1.2	0.2	Yes	837	536	615	36	Yes	No
3	3	1.559	6 h	packed RBC-based	7.414	3.8	1.5	2.8	Yes	3642	1242	339	35	Yes	Yes
4	4	2.111	6 h	packed RBC-based	7.357	2.0	1.0	1.1	Yes	2796	2816	473	25	Yes	Yes
5		2.111	3 h	packed RBC-based	7.017	7.0	1.9	0.6	Yes	2721	2691	547	86	No	
6	5	1.611	5 h 15 min	packed RBC-based	7.194	5.7	1.0	1.0	Yes	1222	1123	559	32	Yes	Yes
7		1.349	6 h	packed RBC-based	6.828	5.7	0.8	0.2	Yes	467	952	342	155	No	
8		2.255	2 h 45 min	packed RBC-based	7.089	12.4	8.4	3.8	Yes	460	1569	465	89	No	

**Table 2:** Summary of complications that occurred within and beyond the 90-day postoperative period. CCI—Comprehensive Complication Index; PNF—Primary Non-Function.

	Complication	Clavien-Dindo	CCI
Patient 1	Diuretics	1	66.1
	Intra-abdominal hematoma in imaging studies	1	
	Prolonged abdominal drainage	1	
	Total parenteral nutrition	2	
	Blood transfusion	2	
	Portal vein kinking—relaparotomy and reanastomosis	3b	
	Dialysis, hemodynamic instability in the postoperative period (vasopressors, prolonged intensive care unit stay)	4b	
Patient 2	Diuretics	1	57.1 (66.3)
	Intra-abdominal fluid collection in imaging studies	1	
	Total parenteral nutrition	2	
	Blood transfusion	2	
	Altered antibiotics regime	2	
	Dialysis	4a	
	Biliary stricture (>90 days following LTx)—endoscopy and bile duct stenting	3b	
Patient 3	Blood transfusion	2	100
	Blood transfusion	2	
	reLTx (PNF)	3b	
	Multiorgan failure (dialysis, hemodynamic instability, acidosis)	4b	
	Death	5	
Patient 4	Multiorgan failure (dialysis, hemodynamic instability, acidosis)	4b	100
	Death	5	
Patient 5	Intra-abdominal fluid collection in imaging studies	1	64.4
	Biliary fistula—relaparotomy and drainage (readmission)	3b	
	Biliary stricture and fistula—endoscopy and bile duct stenting	3b	
	Hemodynamic instability in the immediate postoperative period (vasopressors)	4a	

## Data Availability

The data will be available upon reasonable request.
